# Anatomical spatial-temporal distribution and multivariate risk of acute injuries in elite rugby: a cohort based on prospective surveillance

**DOI:** 10.3389/fphys.2026.1793768

**Published:** 2026-05-08

**Authors:** Zhuo Chen, Xue Wang, Xiaodong Mei

**Affiliations:** 1 School of Media and Art, Tianjin University of Sport, Tianjin, China; 2 School of Sports Training, Tianjin University of Sport, Tianjin, China

**Keywords:** acute injury, anatomical spatial-temporal distribution, logistic regression, risk factors, rugby

## Abstract

**Background:**

Acute injuries are common in rugby and threaten both player health and career longevity. Previous studies often focused on isolated risk factors, while limited research has comprehensively examined the interplay of physiological, environmental, and situational variables.

**Methods:**

Study conducted A cohort study of 40 elite male players from the Tianjin Rugby Team was conducted, monitoring 575 match exposures across 2.5 consecutive seasons (2022–2025). Acute injuries were defined according to international consensus criteria and verified by medical staff. Spatiotemporal distributions (seasonal variation, match stage, playing position, and body site) were analyzed using chi-square and logistic regression. Multivariate models were applied to identify independent risk factors, including demographic, training, and environmental variables.

**Results:**

A total of 143 acute injury events were recorded, with bone and joint injuries most prevalent (48.2%), and the majority classified as moderate-to-severe (78.3%). Injury incidence rate rose significantly across seasons (127.9 per 1,000 player-hours in 2024/25 vs. 43.3 in 2022/23, representing a 1.83-fold increase), and advanced competition stages showed higher risks (OR for finals = 7.06 vs. group stage, p<0.001). GEE analysis demonstrated that the 2023/24 season, semi-finals and finals, forward position, no previous injury history, and higher training load were associated with elevated acute injury risk among rugby players (all P<0.05), while higher temperature served as a protective factor (P<0.001); age, BMI, exercise level, fatigue, and training years showed no significant effects.

**Conclusions:**

Acute injury risk in rugby demonstrates clear spatiotemporal patterns and is strongly influenced by both individual and environmental factors. The model developed provides a practical basis for targeted prevention strategies, including load management, environmental adaptation, and individualized recovery protocols. These findings can serve as a reference for coaches and medical teams at elite Chinese rugby clubs—particularly those adopting training-competition models—to optimize training and competition management.

## 1 Introduction

Rugby is widely regarded as one of the most physically demanding team sports, involving frequent high-impact collisions, rapid offensive-defensive transitions, and complex technical maneuvers. These characteristics contribute to a substantial burden of acute injuries, which may compromise athletic performance, reduce career longevity, and generate considerable medical and economic costs for both teams and society. For instance, nationwide data from New Zealand Rugby indicate that seasonal injuries can lead to significant wage losses and treatment expenditures, with direct medical costs constituting a greater proportion in high-income nations (King et al., 2022; Quarrie et al., 2020). These realities underscore the urgent need for effective injury prevention strategies.

Prior research has identified several key contributors to rugby-related injuries, including a history of previous injury, imbalances in muscle strength, such as defective internal/external rotation strength ratios, and accumulated fatigue (Liu et al., 2012). Epidemiological studies have further emphasized the predominance of lower limb injuries—accounting for 42.5%-58.4% of cases and including particularly hamstring strains and ankle sprains—while also highlighting a notable incidence of clavicle injuries among male players (13.0 injuries/100 player-games) (Quarrie et al., 1996). Additionally, environmental and situational factors, such as playing surface, appropriateness of protective equipment, and specific match phases, such as ball contests, significantly influence injury odds (Bird et al., 1998; Fuller et al., 2007a). It should be clarified that the ‘spatial’ in this study refers to anatomical space (i.e., injury locations and player positions) rather than geographical space, focusing on the distribution characteristics of acute injuries in different body parts and playing positions of athletes.

Despite these insights, most studies have examined risk factors in isolation, limiting broader understanding of how physiological, environmental, and situational determinants interact to elevate injury risk [6]. This gap is particularly evident in the context of Chinese rugby, where Li Yingchao et al. reported a 35.22% injury incidence rate among English rugby players in Chongqing municipality during 2022 (118 cases out of 335 participants) (Gao et al., 2018). Compounding this challenge is the significant variability in recovery trajectories—from one week for mild ankle sprains to six to twelve months for anterior cruciate ligament tears, often accompanied by secondary deficits in decision-making speed and performance upon return to play (McAuley et al., 2022)—which underscores the limitations of solitary intervention measures in minimizing cumulative risk (Fuller et al., 2007b; Bahr et al., 2020; McCrory et al., 2013; Van Gent et al., 2007).

The study aims to address these limitations by systematically evaluating the spatiotemporal dynamics of acute injuries in elite rugby players across multiple competitive seasons. Specifically, we investigate how injury risk varies across temporal (seasonal progression and within-season competition stages) and spatial (injury locations and player positions) dimensions. By uniquely integrating these spatiotemporal patterns with individualized variables (such as fatigue and sleep history), our study analyzes a multivariate Chinese rugby cohort to derive the first discriminative equation for acute injury prediction. By establishing this comprehensive risk model, we aim to provide a theoretical foundation for evidence-based risk management strategies, enhanced injury prevention, optimized load management, and improved competition planning—ultimately supporting player health and athletic performance in rugby.

## 2 Methodology

### 2.1 Study design & data source

The sample for this study was drawn from a single cohort of the Tianjin Rugby Team, comprising 40 male elite athletes. Calculations using G*Power indicate that the sample size meets the minimum requirement. However, it should be noted that the homogeneity of the sample source may be influenced by factors specific to this team, including training style, tactical systems, and regional environment. Therefore, the generalizability of the findings should be evaluated in conjunction with the characteristics of other teams. This investigation constitutes a retrospective cohort study based on prospective injury monitoring. It involves forty male national professional athletes from the Tianjin rugby team who met the inclusion criteria. Of them, 12 are members of the national team representing China at international competitions. The number of acute injury events occurring during the games across 2.5 consecutive seasons (from September 2022 to December 2023 as the first half-season; from March 2024 to June 2025 as the second half-season) was prospectively documented. There are 28 rounds of league games each season, with one game per round for every team. This study included both 15-a-side and 7-a-side rugby formats. Match durations were 80 minutes (two 40-minute halves) for 15-a-side games, and 14 minutes (two 7-minute halves) for 7-a-side games. The same player may have multiple records of injuries or non-injuries in different games.

### 2.2 Sample size and inclusion criteria

Sample size estimation was performed using G*Power 3.1.9.7 for logistic regression (binomial test). Based on preliminary data, we assumed an anticipated odds ratio (OR) of 1.78 for the primary variable of interest (e.g., high training load), a baseline injury probability of 0.15, an alpha level of 0.05, and a statistical power of 0.80. This indicated that a minimum of 563 player-match exposures was required to provide an 80% statistical power to detect an odds ratio of ≥1.78. The final dataset included 575 exposures, meeting the statistical requirement. Inclusion criteria were: (1) male athletes aged 18–28 years; (2) registered professional players; (3) complete exposure and injury data; and (4) no history of severe cardiovascular or cerebrovascular disease. Exclusion criteria included chronic musculoskeletal disorders, recurrent injuries prior to the study period, or incomplete questionnaires.

### 2.3 Data collection procedures

Player data were collected before each competition using the online platform “Questionnaire Star,” supplemented with interviews for clarification. Variables included demographic data (age, height, weight, playing experience, competitive level), injury history, training load, fatigue, and sleep quality. Training load was documented weekly (hours and subjective intensity rating on a 1–10 scale) and validated by cross-checking with coaching staff schedules. Fatigue was assessed using the Fatigue Scale-14 (FS-14), while sleep quality was evaluated with the Pittsburgh Sleep Quality Index (PSQI). Environmental data, including match-day temperature, were also recorded.

Injury data were independently verified by team medical staff and categorized according to type (bone/joint, soft tissue, ligament, concussion, or organ), anatomical site (head/face, upper limbs, torso, lower limbs), and severity (trivial to career-ending, based on time lost). Physicians and physiotherapists confirmed recovery status and return-to-play decisions. (Till et al., 2015) The detailed procedures for data collection and queue construction are provided in the Appendix*.

### 2.4 Recruitment and informed consent procedures

Recruitment channels: Issuing official invitation letters to registered players by means of the Tianjin Rugby Association, specifying the purpose of research, methods of entry, as well as the right of withdrawal.

Content of the informed consent form: It includes the research background, the scope of data collection (health indicators, injury records), privacy protection measures (anonymous processing), the principle of voluntary participation, and contact information.

Signing process: Members of the research team will explain the content of the informed consent form one-on-one to ensure that participants fully understand it, and then they will sign a paper document, which will be archived for future reference.

### 2.5 Exposure and injury rate calculation

The exposure time is based on the actual number of games and appearances of the athletes.

The total number of team rosters, theoretical time on the team or time off the field are not used, only the effective exposure time of actual participation in training/competition is counted.

The injury rate is uniformly expressed in the standard way of exposure hours per 1,000 athletes or playing times per 1,000, ensuring that the overall calculation logic is consistent with that of the seasonal injury rate.

### 2.6 Statistical analysis

All analyses were conducted using SPSS v26.0 (IBM, USA). Categorical variables were summarized as frequencies and percentages, and comparisons were performed using the chi-square or Fisher’s exact tests. Continuous variables were tested for normality and reported as mean ± SD or median (IQR), with independent t-tests or Mann–Whitney U tests applied as appropriate. Principal component analysis was used to test multicollinearity among independent variables. Generalized Estimating Equations (GEE) models were applied to estimate odds ratios (ORs) with 95% confidence intervals (CIs). Model fit was evaluated using the Hosmer–Lemeshow test, and statistical significance was set at p<0.05.

To enhance the robustness of the multifactorial model, multicollinearity tests were conducted on all candidate independent variables before regression analysis. Principal Component Analysis (PCA) and Variance Inflation Factor (VIF) were employed to assess inter-variable correlations. Results indicated that the eigenvalues of all included variables exceeded 0.5, and VIF values were consistently below 5, suggesting no significant multicollinearity. The multivariate logistic regression model was constructed using a forward stepwise approach based on likelihood ratio tests to balance model parsimony and interpretability. Model fit was assessed via the Hosmer–Lemeshow test, complemented by receiver operating characteristic (ROC) curve analysis to evaluate discriminative capability. To test model robustness, sensitivity analyses were conducted by sequentially removing key independent variables to observe changes in regression coefficients. Data missingness was below 3%, and complete-case analysis was applied, as its impact on estimation results was considered negligible.

### 2.7 Variables and strategies

Demographic Characteristics: Age was grouped into quartiles (<21, 21–22, 23–24, and >24 years). Body mass index (BMI) was calculated as weight (kg)/height² (m²) and classified according to Chinese adult reference standards as underweight, normal, overweight, and obese. Athletic Characteristics: Competitive level (elite vs. first-level) and years of rugby participation (<2 years, 2–5 years, ≥5 years) were recorded. Previous injury history (yes/no) was documented through medical verification. Training and Recovery Variables: Training load was assessed weekly using hours of training and subjective intensity ratings (1–10 scale). Fatigue was measured with the Fatigue Scale-14 (FS-14) and categorized as normal (0–9), mild (10–12), or severe (13–14). Sleep quality was evaluated using the Pittsburgh Sleep Quality Index (PSQI), which was classified as very good (0–5), good (6–10), average (11–15), or poor (16–21). Environmental Variables: Ambient temperature on match days was recorded and classified as low (<10 °C), normal (10–25 °C), or high (>25 °C). Spatiotemporal Factors: Season (2022/23, 2023/24, 2024/25), competition stage (group stage [round-robin phase], semi-finals [knockout phase for the top teams], finals [championship-deciding match]),field position (forwards vs. backs), and anatomical site of injury (head/face, upper limbs, torso, lower limbs) were coded as categorical predictors (Van Gent et al., 2007; Till et al., 2015; Mungmunpuntipantip and Wiwanitkit, 2023; Waldén et al., 2022; Buysse et al., 1989; Chen et al., 2024; Pawlikowska, 1993; Zeng et al., 2024). All injury rates expressed per 1,000 player-hours.

## 3 Results

### 3.1 Participant characteristics

A total of 575 player-match exposures were recorded among 40 athletes, resulting in 143 acute injuries over 80,500 exposure hours (1.78 per 1,000 player-hours). The median age was 22 years (IQR: 21–24), and 62.9% of participants competed at the elite level. Previous injury history was common (78.0%) and strongly associated with acute injury occurrence (χ²=16.19, p<0.001). BMI distribution and years of participation were not significantly associated with injury risk. As shown in **Table 1**. The participants’ age structure is bimodal: participants under 21 years of age (23.8%) and those over 24 years of age (15.0%) account for 38.8%. There is no significant relationship between the distribution of grades and injury occurrence (χ² = 4.526, P = 0.033) (West et al., 2023; Lopes et al., 2025).

**Table 1:** Distribution of BMI classification.

BMI classification	Non-injured(%)	Injured(%)	Chi-square test (p)
18.5<x<24	19.44	26.57	0.162
24≤x<28	69.44	65.03	
≥28	11.11	8.39	

Over 90% of participants had≥5 years of sports experience. The chi-square test indicated that there was no significant relationship between injury occurrence and duration of sports (χ² = 1.268, P = 0.561) (Cross et al., 2016; Bjelanovic et al., 2023; Murray-Smith et al., 2023).

### 3.2 Distribution and types of acute injuries

Among the 143 cases of acute injuries, the injury sites mainly involved the knee joint, abdomen, hand, neck and thigh regions, with 78.3% being moderate to severe injuries. According to the nature of the injury, bone and joint injuries were the most common, accounting for 48.2%, followed by soft tissue injuries (34.3%). Concussion and organ injuries were relatively rare, with a combined proportion of less than 5%. From the perspective of anatomical locations, lower limb (42.7%) and upper limb (41.9%) injuries accounted for the highest proportions, while head/face and trunk injuries were relatively less frequent, each accounting for 7.7%. The main causes of injury included site factors, improper recovery measures, non-standard technical movements, lack of agility, and deficiencies in physical fitness and muscle strength; the most common injurious actions were jumping (54 times) and collisions (30 times).

### 3.3 Spatiotemporal distribution

**Figure 1:**
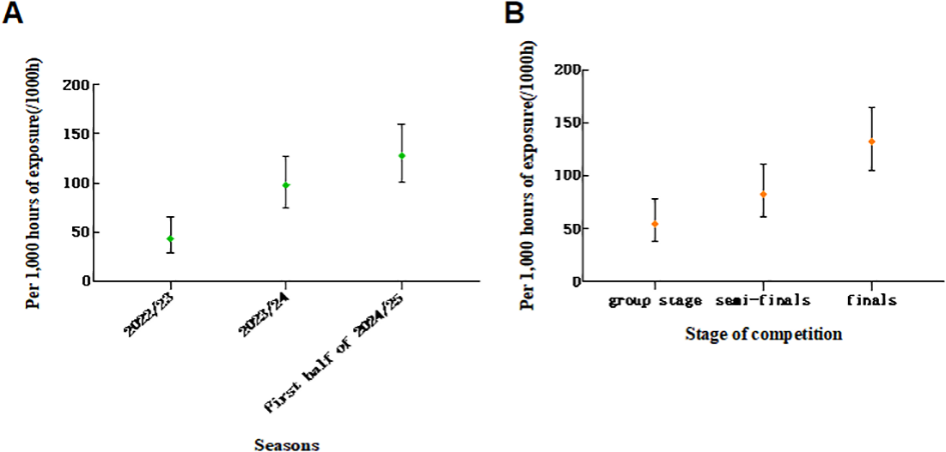
**(A)** Temporal distribution of acute injuries among rugby players during seasons; **(B)** Distribution of acute injuries across different stages of competition. (Note: The 2024/25 season is not a complete competitive season, so the data is for the first half of the season. Error bars represent the 95% confidence intervals).

Seasonal variation: Injury incidence increased across seasons, from 43.3/1,000 exposure hours in 2022/23 to 127.9/1,000 in 2024/25 (χ²=7.64, p=0.022). The risk in 2024/25 was 1.83-fold higher than in 2022/23. As shown in **Figure 1** and **Table 2**.

**Table 2:** Impact of spatiotemporal distribution characteristics on the occurrence of acute injuries among rugby players.

Variable	β value	Standard error	Wald(2	P-value	OR	95%CI
Temporal Distribution Characteristics						
Season						
2023/24(ref=2022/23)	2.459	0.535	21.13	<0.001	11.69	4.097-33.354
2024/25(ref=2022/23)	0.24	0.318	0.57	0.45	1.272	0.681-2.373
Match Phase						
Semi-finals (ref=group stage)	5.832	0.754	59.78	<0.001	341.193	1.096-3.054
Finals (ref=group stage)	3.235	0.383	71.43	<0.001	25.394	4.229-11.798
Spatial Distribution Characteristics						
Field Positions						
Wing forward(ref=forward)	-1.446	0.311	21.61	<0.001	0.235	0.128-0.433
Individual & Training Characteristics						
Individual Factors						
Exercise level = 0 (ref: Exercise level = 1)	0.443	0.361	1.5	0.22	1.557	0.767-3.160
No previous injury (ref = Has previous injury)	2.676	0.523	26.15	<0.001	14.523	5.208-40.498
Fatigue level = 0 (ref = Fatigue level = 2)	1.644	1.177	1.95	0.163	5.176	0.515-52.002
Fatigue level = 1 (ref = Fatigue level = 2)	-0.07	1.231	0	0.954	0.932	0.083-10.414
Training & Environmental Factors						
Training load	0.833	0.336	6.15	0.013	2.301	1.191-4.446
age	-0.249	0.16	2.43	0.12	0.78	0.570-1.067
BMI	0.527	0.287	3.38	0.066	1.693	0.965-2.970
Temperature	-2.239	0.352	40.45	<0.001	0.107	0.053-0.212
Years of rugby training	-0.119	0.098	1.48	0.224	0.888	0.733-1.075

The GEE analysis revealed that the temporal distribution characteristics had a significant impact on the occurrence of acute injuries among rugby players. Taking the 2022/23 season as the reference, the risk of acute injuries among players significantly increased in the 2023/24 season (OR = 11.69, 95% CI: 4.097 - 33.354, P < 0.001), while there was no statistically significant difference in the injury risk in the 2024/25 season (P = 0.45). Regarding the competition stage, compared to the group stage, the acute injury risk was extremely significantly increased in the semi-finals (OR = 341.193, P < 0.001) and the finals (OR = 25.394, P < 0.001), suggesting that the progression of the competition and the increase in the intensity of the confrontation would significantly increase the probability of injury occurrence.

**Figure 2:**
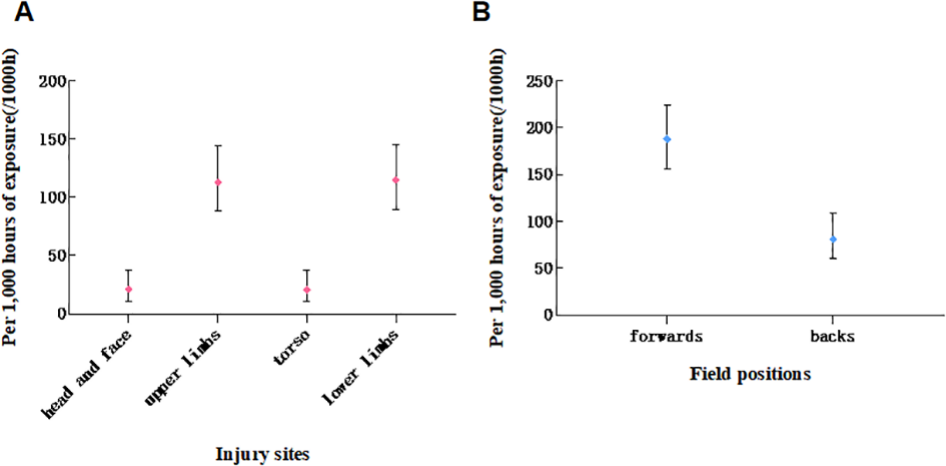
Characteristics of the spatial distribution of acute injuries in rugby players: **(A)** distribution by anatomical injury sites; **(B)** distribution by field positions.

In terms of spatial distribution characteristics, the position on the field is a key influencing factor for acute injuries. Compared with the forward position, the risk of acute injury for flank players was significantly reduced (OR = 0.235, 95% CI: 0.128 - 0.433, P < 0.001), indicating that the forward position, due to its frequent and intense physical confrontations, is a high-risk group for acute injuries, while the injury protection pressure for the flank position is relatively lower (**Figure 2**).

At the individual and training feature levels, multiple factors have a significant impact on acute injuries. Among individual factors, with a previous injury history as the reference, the risk of acute injury for athletes without a previous injury history was extremely significantly increased (OR = 14.523, 95% CI: 5.208 - 40.498, P < 0.001), and there was no significant effect of the level of training and fatigue on the injury risk (P> 0.05). In terms of training and environmental factors, an increase in training load significantly increased the risk of injury (OR = 2.301, 95% CI: 1.191 - 4.446, P = 0.013), while an increase in environmental temperature showed a significant protective effect (OR = 0.107, 95% CI: 0.053 - 0.212, P < 0.001); age, BMI, and training duration had no significant predictive effect on the occurrence of injuries (P > 0.05).

### 3.4 Distribution of basic characteristics of acute injuries

**Figure 3:**
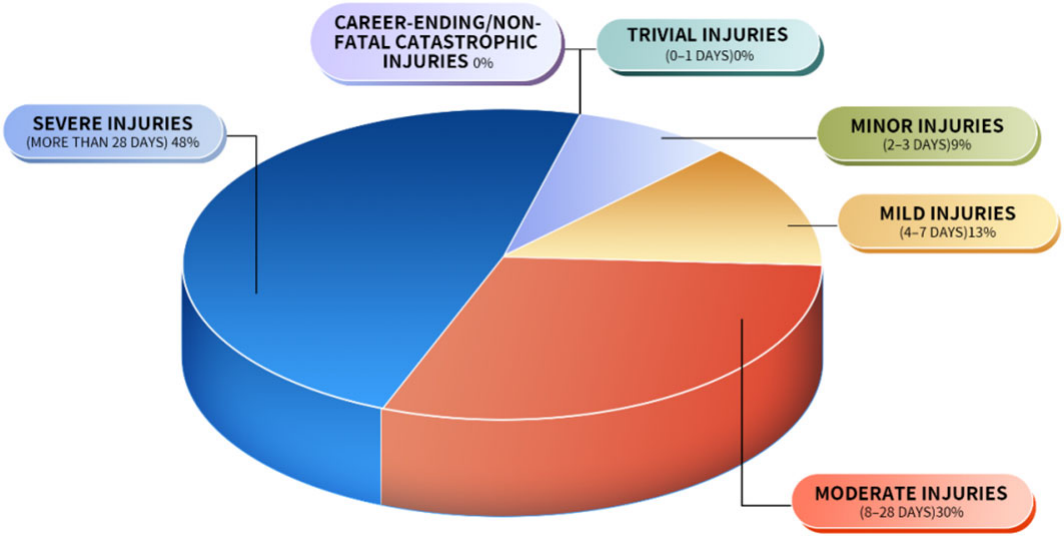
Distribution of severity of acute injuries. Distribution based on 143 injuries over 80,500 player-hours.

**Figure 3** presents the 143 acute injuries rugby players sustained; the majority, 78.32% of cases, were severe. Minor and mild injuries were less common, accounting for 8.39% and 13.29% of the total acute injuries, respectively. No instances of trivial injuries or career-ending/non-fatal catastrophic injuries had been reported(shown in **Figure 3**).

**Figure 4:**
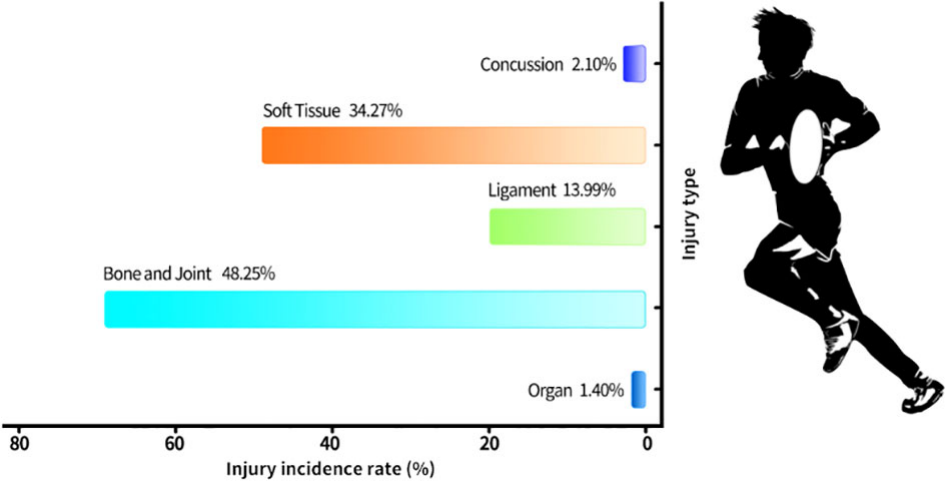
The distribution of the injured body. Distribution based on 143 injuries over 80,500 player-hours.

Regarding injury types, joint and bone injuries were the most prevalent, accounting for 48.25% of acute injuries (Brown et al., 2016; Tranaeus et al., 2024; Hislop et al., 2017). This was followed by soft tissue injuries, which accounted for 34.27%. Other injury types included ligament injuries, concussions, and organ injuries, representing 13.99%, 2.10%, and 1.40% of the acute injuries, respectively (As shown in **Figure 4**).

### 3.5 Univariate analysis

Significant associations with injury occurrence were observed for age group, competitive level, previous injury history, training load, ambient temperature, sleep quality, and fatigue (all p<0.05). No significant effects were found for BMI or years of participation, as shown in **Table 3**.

**Table 3:** Univariate analysis of acute injuries.

Metric	Acute Injuries		χ² value	P-value
	No. (n=432)	Yes(n=143)		
Age (years)			8.859	0.031
<21	103(23.84)	25(17.48)		
21-22	128(29.63)	43(30.07)		
23-24	136(31.48)	39(27.27)		
>24	65(15.05)	36(25.17)		
BMI(kg/m2)			3.641	0.162
≤18.5	0(0)	0(0)		
18.5<x<24	84(19.44)	38(26.57)		
24≤x<28	300(69.44)	93(65.03)		
≥28	48(11.11)	12(8.39)		
Athletic Level			4.526	0.033
Elite	272(62.96)	104(72.73)		
Level 1	160(37.04)	39(27.27)		
Years of Participation in Rugby			1.268	0.561
≤2	4(0.93)	2(1.4)		
2<x<5	45(10.42)	11(7.69)		
≥5	383(88.66)	130(90.91)		
Previous Injury History			16.190	<0.001
No	95(21.99)	10(6.99)		
Yes	337(78.01)	133(93.01)		
Training Load			50.542	<0.001
High	76(17.59)	58(40.56)		
Medium	238(55.09)	62(43.36)		
Low	118(27.31)	23(16.08)		
Temperature			86.869	<0.001
High	24(5.56)	27(18.88)		
Normal	338(78.24)	52(36.36)		
Low	70(16.2)	64(44.76)		
Sleep Quality			34.530	<0.001
Very Good	55(12.73)	23(16.08)		
Good	356(82.41)	93(65.03)		
Average	14(3.24)	11(7.69)		
Poor	7(1.62)	16(11.19)		
Fatigue Scores			21.206	<0.001
None	338(78.24)	84(58.74)		
Mild Fatigue	92(21.3)	57(39.86)		
Severe Fatigue	2(0.46)	2(1.4)		

### 3.6 Multivariate logistic regression

**Figure 5:**
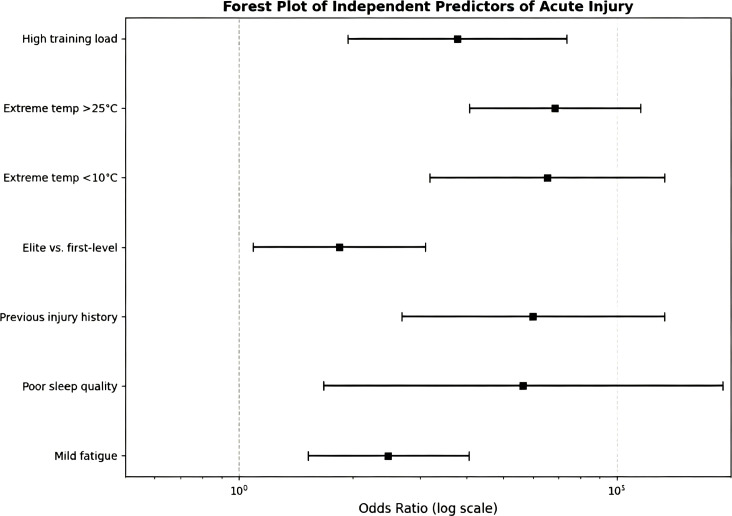
Forecast plot of independent predictors of acute injury.

The independent predictors of acute injury and their corresponding effects on the logarithmic scale are presented in **Figure 5** below.

The regression model demonstrated a good fit (Hosmer–Lemeshow p-value > 0.05), indicating adequate discrimination of risk factors.

This study used binary logistic regression to identify independent risk factors for acute rugby injuries, with principal component analysis confirming no significant multicollinearity (eigenvalues ≥ 0.534). Key findings show that high training loads, extreme temperatures (both high and low), and higher competitive levels (elite athletes face 1.837 times the risk as first-level athletes) significantly increase injury risk. Individual factors like prior injury history, poor sleep quality, and mild fatigue are also closely linked to acute injuries, though severe fatigue shows no statistically significant effect. These factors interact synergistically, indicating that injury prevention strategies should comprehensively address seasonal planning, training load management, and recovery processes (Carron et al., 2024; Leduc et al., 2022; West et al., 2023). As shown in **Table 4**.

**Table 4:** Multivariate logistic regression analysis of acute injuries.

Variable	β value	Standard error	Wald χ2	P-value	OR	95%CI
Training Load						
Medium (ref=low)	0.558	0.316	3.118	0.077	1.747	0.940-3.246
High (ref=low)	1.329	0.337	15.515	<0.001	3.779	1.950-7.321
Temperature			62.706	<0.001		
High (ref=normal)	1.924	0.265	52.571	<0.001	6.849	4.071-11.521
Low (ref=normal)	1.877	0.363	26.659	<0.001	6.531	3.203-13.315
Athletic Level						
Elite (ref=level 1)	0.608	0.268	5.155	0.023	1.837	1.087-3.105
Previous Injury History						
Yes (ref=no)	1.789	0.408	19.191	<0.001	5.982	2.687-13.316
Sleep Quality			21.612	<0.001		
Good (ref=very good)	-0.521	0.333	2.452	0.117	0.594	0.309-1.140
Average (ref=very good)	0.791	0.607	1.700	0.192	2.207	0.671-7.252
Poor (ref=very good)	1.730	0.618	7.852	0.005	5.643	1.682-18.929
Fatigue Symptoms			13.155	0.001		
Mild Fatigue (ref=none)	0.909	0.251	13.142	<0.001	2.481	1.518-4.054
Severe Fatigue (ref=none)	0.102	1.339	0.006	0.939	1.107	0.08-15.271
Constant	-4.534	0.617	54.074	<0.001	0.011	

## 4 Discussion

This study systematically examined the anatomical spatial-temporal distribution (i.e., anatomical space rather than geographical space) and multivariate risk factors for acute injuries among elite rugby players across multiple competitive seasons.

### 4.1 Key findings and contributions

First, the study confirms that acute injuries in rugby are predominantly moderate-to-severe, with bone and joint injuries most common. Importantly, the risk of injury increases substantially during the later stages of the season and in high-stakes competition phases such as finals. These results underscore the cumulative effect of physical load and competitive stress on player vulnerability. While prior studies have often focused on single factors such as match phase or injury type, our research integrates temporal, spatial, and physiological dimensions, offering a more comprehensive understanding of injury dynamics in rugby (Bjelanovic et al., 2023; Murray-Smith et al., 2023; Cousins et al., 2023).

When interpreting the multivariate model, it is critical to consider the magnitude of the odds ratios (ORs) alongside the width of the 95% confidence intervals (CIs) and the number of observed instances. For example, “poor sleep quality” demonstrated a high OR of 5.64, yet its 95% CI was notably wide (1.68–18.93). This wide interval reflects the relatively small number of reported instances in the “poor” category compared to the “good” baseline. Therefore, while poor sleep is a significant risk factor, its precise effect size should be interpreted with caution. Conversely, factors like “high training load” (OR = 3.78, 95% CI: 1.95–7.32) and “previous injury history” (OR = 5.98, 95% CI: 2.69–13.32) had narrower CIs and larger sample distributions, indicating stronger and more robust evidence. Ranked by the reliability of their CIs and the magnitude of their effect sizes, prior injury history, extreme temperatures, and high training load emerge as the primary risk factors in this cohort, followed by sleep quality and fatigue as secondary behavioral predictors.

Second, positional differences were evident: forwards experienced significantly higher injury risk than backs, consistent with their greater involvement in high-impact collisions such as scrums and rucks. This positional disparity underscores the need for tailored prevention strategies, such as enhanced eccentric-strength and stability training for forwards.

Third, the multivariate regression model identified several independent predictors of acute injury, including high training loads, extreme temperatures, elite competition level, prior injury history, poor sleep quality, and mild fatigue. The combination of physiological, environmental, and behavioral factors highlights the multifactorial nature of injury risk. By quantifying these effects, our model provides an evidence-based framework for individualized injury prevention and early warning systems.

### 4.2 Comparison with previous literature

Our results align closely with prior literature across sports. The finding that high training load significantly increases injury risk (OR = 3.78) is consistent with (Cross et al., 2016), who reported that spikes in accumulated workload elevate injury susceptibility in rugby union. Similarly, our data confirmed that a history of previous injury is a major predictor (OR = 5.98), echoing the classic findings of (Hägglund et al., 2006) regarding the high risk of recurrent injuries in elite team sports. While existing univariate studies have highlighted environmental and behavioral factors individually, our multivariate model demonstrates their synergistic impact, confirming that extreme temperature and poor sleep quality are independent predictors of injury (Clemente et al., 2021; Tranaeus et al., 2024). However, this study extends existing knowledge by demonstrating that environmental extremes—both heat and cold—are comparably potent risk factors. The integration of sleep quality and fatigue assessments also advances current models, as these recovery-related variables are often underrepresented in rugby injury surveillance.

### 4.3 Practical implications

From an applied perspective, these findings support the implementation of multifactorial monitoring systems in elite rugby. Regular assessment of training load, sleep quality, and fatigue can enable early identification of at-risk players. Coaches and medical teams should adjust workloads, introduce mandatory rotation during congested schedules, and employ targeted interventions, such as heat-adaptation protocols or recovery-focused training. Moreover, positional demands necessitate role-specific injury prevention programs. Regarding potential biases, the Tianjin rugby team’s training intensity, match scheduling, and athlete selection criteria may differ from those of other domestic teams. This could make it difficult to generalize certain findings to all Chinese rugby teams directly.

The findings provide direct practical guidance for injury prevention and risk management in elite rugby. Training load, as one of the most critical modifiable factors, shows a significant positive correlation with acute injury occurrence. This suggests that during the mid-to-late season and periods of dense scheduling, the cumulative risk for athletes should be reduced through scientifically phased training and load monitoring. An integrated monitoring system combining training duration, subjective intensity ratings, and recovery indicators facilitates early identification of high-risk individuals.

Regarding environmental factors, the impact of extreme heat and cold on acute injuries is comparable to individual physiological factors. This underscores the need to consider environmental adaptation strategies before competitions and training sessions thoroughly. Examples include extending the dynamic warm-up duration in cold conditions and implementing heat-acclimatization training and hydration management in high-temperature environments.

Additionally, forwards exhibit significantly higher injury risks than defenders, supporting position-specific prevention protocols such as enhancing collision tolerance, neck-shoulder stability training, and post-match recovery management. Sleep quality and fatigue status, as measurable behavioral variables, serve as early warning indicators, enabling coaches and medical teams to optimize decisions without increasing training load.

### 4.4 Limitations and future directions

Several limitations should be acknowledged. It is declared as a pilot study based on a single team with a relatively small sample size, which may limit generalizability, and multi-center validation is recommended. Training load was self-reported and not complemented by objective biomarkers such as GPS data, lactate concentration, or heart rate variability. Additionally, injury surveillance focused on match play, excluding training-related injuries that may contribute to overall risk. Future research should address these gaps by incorporating larger multi-center cohorts, integrating wearable monitoring technologies, and extending surveillance to both training and competition contexts.

Although this study is based on a sample from a single elite rugby team, several core findings align closely with international rugby injury research, including a high proportion of moderate-to-severe injuries, increased injury risk for forward positions, and the significant impact of prior injury history and training load. This partially supports the potential generalizability of the findings to high-level rugby environments.

Concurrently, the training-competition model in Chinese rugby exhibits certain unique characteristics, such as centralized training arrangements and phased intensive competition schedules. As established in elite sports literature, such dense schedules and the resulting cumulative fatigue significantly amplify the overall injury risk (Gabbett, 2016). Therefore, future research should validate the risk model developed in this study across multiple-center settings, varying competitive levels, and regional teams to enhance its applicability and robustness.

## 5 Conclusion

This study demonstrated that acute injuries among elite rugby players follow distinct spatiotemporal patterns, with risks heightened during later competitive stages, finals, and in players occupying forward positions. Bone and joint injuries were most common, and the majority were of moderate-to-severe grade, underscoring the heavy burden of injury in this sport.

From a practical perspective, the results support the development of individualized prevention strategies and monitoring systems. Training loads should be carefully periodized, recovery protocols strengthened, and environmental adaptation strategies adopted. Sleep and fatigue monitoring can provide early warning signals, while position-specific programs may help address the elevated risks faced by forwards.

## Data Availability

The raw data supporting the conclusions of this article will be made available by the authors, without undue reservation.

## Appendix*

**Details of data collection and the complete process**

Prior to each competition, participant data were collected using the online application “Questionnaire Star.” The collected data included: age, height, weight, athletic level, years of rugby participation, history of sports-related injuries, training load, temperature, sleep quality, and fatigue levels. All 40 participants were individually explained by the research team prior to completing the questionnaire to confirm that they understood the meaning of the questions and the mode of completion. For the junior players aged 18 - 20 (15 participants, representing 37.5% of the total), a 10-minute face-to-face clarification was provided. The technique of providing examples (e.g., “Did you feel too tired as a result of training over the previous week? Tick ‘Yes’ or ‘No’”) was used to ease the difficulty of understanding. Self-reported duration and rating of training load: Participants completed a training record every week, including: Duration: precise to the hour (e.g., “Trained 12 hours over the week”). Intensity rating: subjective 1–10 rating (1 point = easy, 10 points = extreme). The research team verified the training schedules with the coaches every month to ensure that self-reported durations aligned with the actual schedules.

Injury data were recorded by the medical staff responsible for diagnosing and treating injuries within each team. For Return-to-Play (RTS) data, the decision on when athletes could return to competition was made by the medical staff, including treating physicians and physiotherapists, who evaluated the athletes’ recovery. These RTS data were confirmed upon medical staff approval and the athlete’s inclusion on the official match roster. Notably, these data were not self-reported by athletes but were obtained directly from the medical professionals involved in the rehabilitation process and were verified by the research team.

Injury data were recorded by medical staff responsible for diagnosing and treating injuries within each team. These data encompassed injury characteristics (type, location, site of occurrence, and severity) and the phase of the game during which the injury occurred. All injuries were classified according to the definitions established by the Rugby Injury Consensus Group (RICG)[9]and the 2020 statement of the International Olympic Committee[10]. The severity of each injury was categorized based on the number of days required for recovery: trivial injuries (0–1 day), minor injuries (2–3 days), mild injuries (4–7 days), moderate injuries (8–28 days), severe injuries (exceeding 28 days), and career-ending/non-fatal catastrophic injuries[9].

To ensure the accuracy of the injury data, the diagnosis and classification were double-checked by at least two medical staff members, and the recorded data were cross-verified by the research team. Additionally, researchers consistently tracked athletes who sustained acute injuries to determine the date of their return to competition and to confirm the accuracy of recovery assessments.

Furthermore, all injury data were assessed for consistency with the athletes’ prior injury history, training loads, and physical condition at the time of injury. This validation process was crucial for maintaining the integrity and accuracy of the data throughout the study.

Currently, the incidence rate of injuries across all sports is commonly expressed per 1,000 hours of exposure. Exposure time was calculated by coaching and technical staff based on match records and was subsequently verified by the research team. The exposure hours for each game correspond one-to-one with the injury events that occur in that game, ensuring the accuracy of the incidence rate calculation. To more accurately measure the actual time spent at risk, the formula for calculating the incidence rate of acute injuries during exposure time is shown in the context formula.
